# Influences on change in expected and actual health behaviors among first-year university students

**DOI:** 10.1080/21642850.2023.2174697

**Published:** 2023-02-04

**Authors:** Chrys Gesualdo, Martin Pinquart

**Affiliations:** Department of Psychology, Philipps University Marburg, Marburg, Germany

**Keywords:** Food consumption, physical activity, alcohol, health behavior expectations, Covid-19

## Abstract

**Background:**

First-year students often adopt health risk behaviors during their first semester such as increased consumption of unhealthy food, decreased physical activity, and increased alcohol use. Expectations, social tie's efforts to motivate behavior, and coresidence with parents can influence said behaviors.

**Aims:**

We assessed how students’ health behaviors and expectations change over the first semester, and how the aforementioned factors influence the maintenance or change of behavior and expectations.

**Methods:**

A longitudinal survey design was implemented. A total of *N* = 163 German first-year students (81% female; 18% male; 1% non-binary; *M*_age_ = 21.20, *SD* = 2.66) completed online questionnaires, including the NCHRBS and AUDIT, during the Covid-19 pandemic at the beginning (November 2020) and after the end (May 2021) of their first semester.

**Results:**

Current and expected food consumption and physical activity became healthier over time. The current and expected number of drinks consumed per month increased. Change in expectations for physical activity, number of drinks and binge drinking were predicted by the initial respective behavior. The number of drinks and expected physical activity became unhealthier in relation to reported initial parental influence to drink and to be physically inactive. Moving out of the parental home predicted an increase in current and expected number of drinks and in current and expected binge drinking. These effects of moving out were not mediated by perceived parental or peer influence.

**Conclusions:**

Interventions should target these behaviors and expectations during the first semester and address parental influence on physical activity and alcohol use.

Previous studies have consistently demonstrated that a large percentage of first-year students adopt health deteriorating behaviors such as increased consumption of unhealthy food (i.e. low consumption of fruits, vegetables and dietary fiber and high consumption of saturated fats, sodium, and refined sugar; Maillet & Grouzet, 2021), decreased physical activity (Deng et al., 2021), and increased alcohol use as well as binge drinking, particularly during the first semester (Romm et al., 2020). It is well established that consuming healthy food as well as regular engagement in physical activity and limiting alcohol use serve as protective factors against conditions such as type 2 diabetes, cardiovascular diseases, and certain types of cancer (Hilger et al., 2017). A meta-analysis of weight gain in first-year university students found that most students gained weight during the first semester of the first year (Vadeboncoeur et al., 2015). Increased consumption of unhealthy food and decreased physical activity appear to be significantly correlated with the observed weight gain during the first semester (Deng et al., 2021). Furthermore, a large percentage of students consume critically high amounts of alcohol during their first semester (Riordan & Carey, 2019). While scant research exists investigating health behaviors of first-year German university students, some evidence exists indicating deficient consumption of healthy foods (Keller et al., 2008), insufficient physical activity (Diehl & Hilger, 2016; Keller et al., 2008), as well as risky alcohol consumption and binge drinking (Helmer et al., 2010; Keller et al., 2008) among this population.

Risky health behaviors have been linked to university students, and the college environment provides a context associated with engagement in said behaviors (Romm et al., 2020), which may influence students’ behavioral expectations about their time at university. As such, the transition to university marks a vulnerable period for the development of health risk behaviors that will consequently negatively impact future health and personal outcomes (Maillet & Grouzet, 2021). As students’ health behaviors are expected to worsen during their first semester, understanding factors that influence behavioral change is pivotal to battle risky behaviors and prevent undesirable health outcomes among students.

## Influences on health behaviors

### Expectations

The Theory of Planned Behavior (TPB; e.g. Bosnjak et al., 2020) and the ViolEx model (Rief et al., 2015) suggest that expectations are important determinants of future behavior. In TPB, the attitude component refers to the expected consequence of a behavior and the control component is usually measured via self-efficacy (the expectation of being able to show a particular behavior). For instance, individuals who believe that they can be active and who expect favorable outcomes from physical activity are more likely to adopt and maintain said behavior (Anderson et al., 2006). In the case of discrepancies between the present state and related expectations, individuals may change expectations in the direction of the present state or change the present state in the direction of the expected state (Rief et al., 2015). As such, expectations change can be fostered by past and present experiences and behavior. For instance, previous experiences with programs that promote healthy food consumption as well as physical activity influence future outcome expectations and future behavior (Herriot et al., 2008). Thus, it is plausible that expectations about future behavior influence alterations in behavior to match expectations, and that previous or current experiences reinforce expectations.

The Violated Expectations (ViolEx) Model proposes that, in the face of evidence disconfirming initial situation-specific expectations, maintenance or change of expectations depends on the particular psychological process to cope with disconfirmed expectations occurring within an individual (Panitz et al., 2021). Three strategies for coping with expectation violation are specified in the ViolEx Model, namely: immunization, assimilation, and accommodation (Panitz et al., 2021). Immunization refers to the minimization of the impact of expectation-disconfirming evidence and has been associated with the persistence of expectation (Panitz et al., 2021). Assimilation occurs when an individual searches for or produces future expectation-confirming evidence and has been associated with persistence of expectations (Panitz et al., 2021). Accommodation occurs when individuals update their expectations in the direction of the experienced outcome, thus indicating expectation change (Panitz et al., 2021). These processes have not yet been applied in longitudinal research on expectations about health behaviors.

Discrepancies between expected and actual behavior also relate to the intention-behavior gap, a phenomenon where individuals intend to behave in a particular way but finally do not show the intended and expected behavior. Unexpected events or experiences could lead (amongst others) to said discrepancy. In the case where expectations refer to one's intended behavior, expectation violations and the gap between intention and final behavior tend to occur together. Nonetheless, expectations and expectation violations do not always refer to one's intended behavior. Furthermore, it has been demonstrated that changes in expectations may lead to behavior change. A meta-analysis examining the effects of expectancy challenge (EC) interventions among college students in the United States and Europe showed that a reduction of student's positive alcohol expectations achieved through participation in the EC was related to a significant decrease in positive alcohol expectations (Gesualdo & Pinquart, 2021).

### Social tie’s efforts to motivate behavior

The ViolEx model further posits that social influences can affect an individual's expectation updating (Panitz et al., 2021). Close social ties’ (i.e. parents, romantic partner, peers) attempts to influence an individual's behavior may also play a significant role in health behavior (Kaseva et al., 2017) and expectation (Panitz et al., 2021) change. Information provided by social ties and their attempts to encourage behaviors may be mechanisms through which individuals adjust their behavior and health-behavior-related expectations (Legros & Cislaghi, 2020; Panitz et al., 2021). Nevertheless, little empirical research has addressed the role of social influences on change of health-behavior-related expectations.

### Living with parents and health behavior change

According to the ViolEx model, characteristics of the environment (e.g. living arrangement) can influence an individual's expectations (Panitz et al., 2021). In line with this notion, Deliens et al. (2014) found that students perceive that living with their parents influences their expectations about their future behavior. Moving out of the parents’ residence often facilitates health demoting opportunities as living with parents usually provides a structure that enables healthy behaviors and inhibits unhealthy behaviors (Jones et al., 1992). Living with parents increases the likelihood that students will consume more healthy foods (Maillet & Grouzet, 2021) and less alcohol (Jones et al., 1992) over time. Evidence for physical activity is mixed as some studies report that students who live with their parents are more physically active than those who do not (Fan et al., 2019), whereas other studies report the opposite (Jones et al., 1992).

## The present study

As several health behavior patterns are formed during the first year at university (Maillet & Grouzet, 2021), understanding risky health behavior determinants among students during this vulnerable period is fundamental for developing appropriate structured support to prevent adverse outcomes on future health. Although previous studies have demonstrated that expectations are likely to influence behavior (Rief et al., 2015), the role of expectations on health behavior change during the first year has not been extensively investigated in previous studies, and investigations concerning the maintenance or change of health-behavior-related expectations are limited and mostly refer to alcohol use. Moreover, the process that relates to the maintenance or change of health-behavior-related expectations has not been previously assessed among first-year students. Findings on this topic will inform future health promotion efforts on how to foster healthy expectations and behavior among first-year students. Furthermore, the influence of parents’, partner's, and peers’ attempts to motivate behavior on expectation maintenance or change has not been simultaneously addressed. Findings on this matter can highlight which social ties have stronger motivating influence on first-year students’ health behaviors. Finally, the relationship between coresidence with parents and health-behavior-related expectation change as well as behavior change has not been broadly investigated in the existing literature, particularly regarding physical activity. Based on the VioleEx Model (Panitz et al., 2021), the present study addressed the aforementioned gaps by analyzing longitudinal data to investigate change in behaviors and in expectations.

We first assessed how first-year students’ health behaviors (i.e. food consumption, physical activity, alcohol use) and respective expectations change over the first semester. Hypothesis 1 states that first-year students’ health behaviors and expectations will become unhealthier throughout the first semester. Moreover, we assessed whether present health behaviors predict expectation change over time and whether present expectations predict change in health behaviors, thus leading to a decline of discrepancies between health behavior and related expectations. Hypothesis 2 assumes behavior change in the direction of the initial expectation. Hypothesis 3 proposes that behavior at the first point of measurement will predict change in expectations (expectations become more aligned with the actual behavior). In addition, we investigated whether coping with expectation disconfirmation relates to expectation change. Hypothesis 4 states that participants with higher initial accommodation scores will show more expectation change while participants with higher immunization and assimilation scores will present less expectation change. Furthermore, we investigated whether social ties’ efforts to influence health behaviors are associated with change in health behaviors and related expectations. Hypothesis 5 posits that participants will change their behaviors and expectations to reflect their social ties’ (parents’, peers’, romantic partner's) influence. Lastly, we investigated whether changes in health behaviors and expectations depend on whether students moved out of their parents’ home. Hypothesis 6 proposes that participants who had moved out of their parents’ home at the start of their study will present stronger increases in unhealthy behavior and related expectations than participants who did not move out of their parents’ home, and that this effect is, at least in part, mediated by differential influences of parents and peers. Peers might have a stronger influence than parents, particularly regarding alcohol use, if participants no longer live with their parents.

## Methods

A longitudinal survey design was implemented. The study was approved by the Ethics Committee at the University of Marburg, Germany (file number 2020-79k). Data collection took place via online, anonymous questionnaires during the Covid-19 pandemic at two times of measurement, namely at the beginning (November 2020) and after the end (May 2021) of the first semester. Approximately 500 first-year students in Germany who were at least 18 years old (above the legal drinking age) were recruited via e-mails with a link directing them to the questionnaires. Out of the recruited participants, *n* = 212 responded at time 1 out of which *n* = 166 provided data at the 6-month follow-up. Incomplete questionnaires as well as outliers were excluded from the analysis.

Participants completed the questionnaires after reading study-related information and granting consent. Participants needed a maximum of 30 minutes to complete the questionnaires. University credit points or participation in a gift card raffle were offered as compensation.

### Measures

#### Sociodemographic characteristics

Demographic questions were administered during both points of measurement and assessed gender (male, female, non-binary), age, and whether participant’s hometown is in Germany or abroad (specific ethnic background was not assessed to prevent reidentification).

#### Partner status and residence

In addition, we assessed whether participants have a partner or not and whether they moved out of their parents’ home or not to attend university for hypothesis testing.

#### Food consumption

The seven-item National College Health Risk Behavior Survey (NCHRBS; Douglas et al., 1997) was administered at both points of measurement to investigate how many times a day in the past month participants consumed unhealthy food. Two additional items following the NCHRBS's format designed for our study were also included to assess further food often consumed by students (i.e. pizza as well as sweets and chocolate). All nine items included a Likert scale response format of 0 times a day, 1 time a day, 2 times a day, and 3 or more times a day. Higher scores represented higher levels of unhealthy eating. The scale showed acceptable reliability for our data with a Cronbach's coefficient of α = .60 for the first point of measurement and of α = .67 for the second point of measurement. Additionally, rephrased versions of the nine items were also administered at both points of measurement to assess expected food consumption (e.g. on average, during this semester, how often per day do you expect to eat fruits?). Expected food consumption items showed acceptable consistency of α = .70 for the first measurement and of α = .77 for the second measurement.

#### Physical activity

Four items of the NCHRBS's physical activity scale (Douglas et al., 1997) were administered at both points of measurement to assess how many times a week in the last month participants performed physical activity (i.e. cardio, strengthening, stretching, and walking or cycling). The items included a Likert scale response format ranging from 0 to 7 times a week. Scores were reversed so that higher scores denoted higher levels of physical inactivity. The NCHRBS physical activity scale has shown excellent test-retest reliability and validity indices similar to other self-report physical activity questions (Dinger, 2003). The scale has also been used successfully in empirical research among university students of various backgrounds to assess physical activity among college students (e.g. Ajibade, 2011). For our study, the scale showed an acceptable internal consistency of α = .76 for the first point of measurement and of α = .78 for the second point of measurement. Furthermore, rephrased versions of the four items were also administered at both points of measurement to assesses expected physical activity (e.g. on average, during this semester, how many days a week do you expect to walk or ride a bike for at least 30 minutes?). Expected physical activity items showed consistency of α = .76 for the first and of α = .77 for the second measurement.

#### Alcohol use

Three items from the Alcohol Use Disorders Identification Test (AUDIT; Saunders et al., 1993) were adapted to assess drinking behavior and were administered at both points of measurement. The first (i.e. how many days during the past month did the participant consume alcohol) and second (i.e. how many standard drinks did a participant drink on a drinking occasion) items had a free input response format and both results were multiplied to calculate the number of drinks consumed by participants in a month. The third item assessed binge drinking by asking how often participants drink five (for males), four (for females) or more standard drinks in a drinking occasion (National Institute on Alcohol Abuse and Alcoholism, 2012), with a Likert scale response format ranging from never, less than monthly, monthly, to weekly. As there are no established practices to assess binge drinking among sexual minority groups, the male threshold was used to assess binge episodes in non-binary individuals based on findings suggesting that a higher proportion of non-binary individuals are assigned a male sex at birth in comparison to binary individuals (Todd et al., 2019). Rephrased versions of the three items were also administered at both measurements to assesses expected drinking behavior (e.g. how many days per month do you expect to consume alcohol during this semester?).

#### Coping with expectation violations

To assess individual differences in coping with disconfirmed expectations, the ViolEx-Questionnaire (Pietzsch et al., 2020) was administered at the first point of measurement. The twenty-six-item questionnaire consists of three scales, each of which assessed an individual coping strategy (i.e. accommodation, assimilation, and immunization). The assimilation scale (e.g. I am actively committed to ensuring that my expectations come true) consisted of 10 items, while the accommodation (e.g. I adjust my expectations when a situation requires it) and immunization scales (e.g. If I experience something that doesn't fit my expectations well, then I see this as an exception) consisted of 8 items each. Items addressed the extent to which a statement applied to an individual with a four-point rating scale ranging from 1 = strongly disagree to 4 = strongly agree. Higher scores denoted higher use of a particular strategy. Cronbach's alpha was excellent for the assimilation *α* = .87, the accommodation *α* = .85, and the immunization scale *α* = .86.

#### Social ties’ efforts to encourage health risk behaviors

Nine items inquiring about social ties’ efforts to motivate health demoting behaviors were developed and were administered at the first point of measurement (see supplementary material S1). Single items assessed how often each social tie motivates the participant to eat unhealthy food, to be physically inactive, and to consume alcohol (e.g. how often do your peers encourage you to consume unhealthy foods?). Motivation deriving from the parental dyad and the peer group was assessed (i.e. three items for parents and three items for peers). Participants who initially stated that they have a partner completed three additional questions. Responses ranged from 4 = very often to 1 = never, and higher scores denoted more frequent encouragement for unhealthy behaviors.

### Statistical analysis

IBM SPSS Statistics version 27 and Lisrel 8.8 were used for data analysis. A power analysis specified a minimum sample size of *N* = 108 to identify small effects with 80% power at an alpha level of .05. A self-generated code by participants was used to link corresponding surveys of both points of measurement. To test for systematic loss to follow-up, participants who were lost and who stayed in the study were compared regarding demographics and outcome variables. Categorical variables were analyzed with Chi^2^ tests and continuous data with *t*-tests. Normality tests were conducted. Listwise deletion was used for handling missing data. An Alpha level of 5% was implemented to identify significant results. The first hypothesis was tested using ANOVAs with repeated measures including the means of current and expected behaviors, respectively, at both time points. Structural equation models were implemented to test the second and third hypotheses. The fourth and fifth hypotheses were tested using linear regressions with behavior and expectations at the second point of measurement as outcome variables, and initial behavior and expectations as well as scores in the ViolEx questionnaire and social ties’ influence on health behaviors as predictors. The sixth hypothesis was tested using regression analyzes (health behaviors and expectations), as well as to assess whether moving out of the parental home predicts change in health behaviors and expectations, as well as using structural equation models for testing mediating effects. Age and sex were included as covariates in all analyzes.

## Results

### Sociodemographic characteristics

A total of *N* = 208 first-year students agreed to participate in our study. From those, *n *= 166 participants completed the questionnaires at both points of measurement out of which *n* = 3 were excluded from our analysis as outliers due to reported extreme drinking scores. The final sample consisted of *N* = 163 first-year students (81% female; 18% male; *M*_age_ = 21.20, *SD* = 2.66) out of which *n* = 155 (95%) reported that their hometown is in Germany, *n* = 66 (41%) reported having a partner, and *n* = 125 (77%) moved out of their parents’ home. As participants had already been, on average, *M* = 25.98 (*SD* = 10.45) days in the first semester at the first data collection point, the first assessment referred to the first days at university and the longitudinal study assessed change in health behaviors and expectations during the first semester (i.e. we did not assess changes from pre-college time to time at college). Participants who dropped out did not significantly differ to the remaining participants regarding demographic variables and initial health behaviors (see supplementary material S2). Bivariate correlations between all model variables are provided in the supplementary material (S3). No significant effects of age and sex were found in hypothesis testing.

### Longitudinal behavior/expectation change

Consumption of healthy food, participation in physical activity, and the number of drinks consumed per month increased over time (see Table 1). As the number of drinks per month was based on information about the number of days they consumed alcohol and the mean number of drinks per day, we also checked whether both numbers changed. Only the former number increased. In addition, no significant changes were found regarding current level of binge drinking. Furthermore, expected consumption of healthy food, expected participation in physical activity, and expected number of drinks increased during the first semester. Expectations about future binge drinking did not change significantly.

**Table 1.** Change in Health-Related Expectations and Behavior Between Time 1 and Time 2.

	*M_1_*	*SD_1_*	*M_2_*	SD*_2_*	*F*	*df*	*p*
**Behavior**							
FC	19.63	2.76	15.72	2.57	229.22	162	<.01
PA	22.85	5.79	13.88	5.96	149.82	162	<.01
ND	11.00	14.44	25.03	27.68	47.75	162	<.01
Frequency AU	3.98	4.54	10.09	8.87	84.27	162	<.01
Quantity AU	2.40	2.69	2.15	1.85	1.37	162	.25
BD	2.01	.94	1.97	.90	.21	162	.65
**Expectation**							
FC	19.23	2.72	15.50	2.63	184.69	162	<.01
PA	21.34	5.68	15.91	5.84	57.76	162	<.01
ND	11.01	13.15	22.51	30.68	22.85	162	<.01
Frequency AU	3.93	3.87	8.37	8.67	47.20	162	<.01
Quantity AU	2.34	2.58	2.16	1.87	.71	162	.40
BD	1.90	.93	1.89	.85	.64	162	.94

Note*. N = 163.* FC = food consumption; PA = physical activity; ND = number of drinks consumed per month; BD = binge drinking; AU = alcohol use; *M_1 _*= mean score at time 1; SD_1_*_ _*= standard deviation at time 1; *F* = test of change in health behavior and health-related expectations between both times of measurement; *df* = degrees of freedom.

### Longitudinal interplay of behavior and expectations

**Figure 1.**
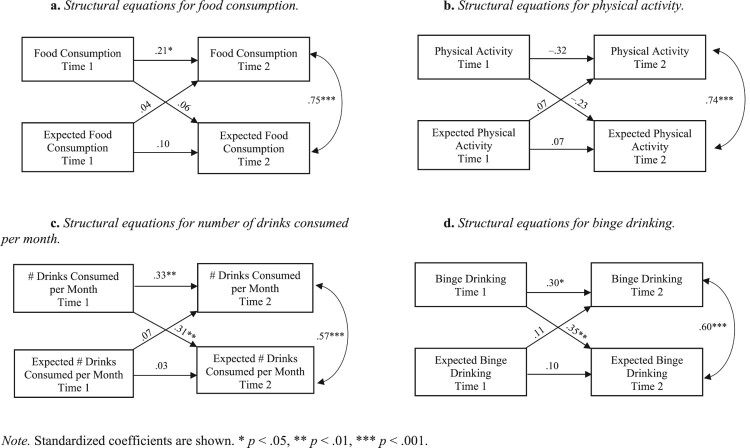
(a) Structural equations for food consumption. (b) Structural equations for physical activity. (c) Structural equations for number of drinks consumed per month. (d) Structural equations for binge drinking. *Note.* Standardized coefficients are shown. * *p* < .05, ** *p* < .01, *** *p* < .001.

Cross-lagged (autoregressive) panel models were computed that assess whether initial expectations predict change in related health behavior and whether initial behavior predicts expectation change over time. As shown in Figure 1(a–d), food consumption, number of drinks consumed per month, and binge drinking showed some correlational stability. In contrast, we found a non-significant negative association between reported physical activity at the first and second point of measurement. Associations between expectations at the first and second point of measurement were not significant, while reported present and expected future activities were concurrently correlated. Furthermore, initial expectations about food consumption, physical activity, number of drinks consumed per month, and binge drinking did not predict change in the respective behavior. Change in expected numbers of drinks and in binge drinking were predicted by the initial levels of these behaviors. In contrast, higher initial physical passivity predicted a stronger *decline* in the related expectation over time, and initial food consumption did not predict change in the related expectation over time.

### Coping with expectation violations

Higher immunization predicted less change in expectations about physical activity, and higher assimilation significantly predicted greater increases in expectations about future physical activity (see Table 2). However, participants with higher initial accommodation did not show greater expectation change.

**Table 2.** Associations of coping with expectation disconfirmation with students’ change in expected health behavior (regression analysis).

	Expected food consumption (T_2_)			Expected physical activity (T_2_)			Expected # of drinks consumed monthly (T_2_)			Expected binge drinking (T_2_)		
	*B*	*SE B*	β	*B*	*SE B*	*β*	*B*	*SE B*	*β*	*B*	*SE B*	*β*
HB at T_1_	.15	.08	.15	–.25	.08	–.24^b^	.46	.18	.20^a^	.35	.07	.38^b^
Immunization	–.06	.05	–.11	–.27	.12	–.21^a^	–.29	.62	–.04	–.03	.02	–.13
Assimilation	.07	.05	.13	.22	.10	.19^a^	–.35	.53	–.06	–.01	.01	–.04
Accommodation	.02	.05	.04	.06	.10	.05	.01	.55	.01	–.01	.01	–.01
*R*^2^		.04			.11^b^			.05			.17^b^	

Note. *N* = 163. HB = health behavior. T_1_/T_2_ = first/second point of measurement. *B* = unstandardized regression coefficient. *SE* = standard error. *β* = standardized regression coefficient. HB at T_1_ corresponds to the behavior represented in each column. ^a^*p* < .05, ^b^*p* < .01.

### Social ties’ influence on behavior/expectation change

Initial perceived parental attempts to promote alcohol use and physical inactivity predicted an increase of this behavior and expectation, respectively, over time (see Table 3). No significant results were found regarding partners and peers.

**Table 3.** Associations of social tie's efforts to motivate unhealthy behaviors with students’ change in current and expected health behavior (regression analysis).

	Current food consumption (T_2_)			Current physical activity (T_2_)			Current # of drinks consumed monthly (T_2_)			Current binge drinking (T_2_)		
	*B*	*SE B*	*β*	*B*	*SE B*	*β*	*B*	*SE B*	*β*	*B*	*SE B*	*β*
HB at T_1_	.28	.11	.31^a^	–.24	.14	–.21	.68	.20	.38^b^	.20	.13	.21
Parents’ Influence UB	–.13	.54	–.03	3.19	1.65	.25	14.69	6.29	.29^a^	.26	.29	.13
Partner's Influence UB	–.22	.46	–.06	.01	1.30	.01	–3.06	3.89	–.11	.22	.18	.19
Peers’ Influence UB	–.09	.46	–.03	.37	1.56	.03	–6.24	3.37	–.24	–.20	.16	–.19
*R*^2^		.11			.10			.33^b^			.13	

	Expected food consumption (T_2_)			Expected physical activity (T_2_)			Expected # of drinks consumed monthly (T_2_)			Expected binge drinking (T_2_)		
	*B*	*SE B*	*β*	*B*	*SE B*	*β*	*B*	*SE B*	*β*	*B*	*SE B*	*β*
Expected HB at T_1_	.19	.12	.19	–.25	.14	–.21	.15	.28	.07	.14	.12	.15
Parents’ Influence UB	.45	.57	.10	3.29	1.52	.28^a^	14.89	9.21	.24	.39	.26	.22
Partner's Influence UB	–.72	.49	–.19	–.60	1.22	–.07	–1.78	5.75	–.05	.17	.16	.16
Peers’ Influence UB	–.47	.49	–.13	1.39	1.45	.13	–4.65	5.01	–.14	–.12	.14	–.13
*R*^2^		.11			.13			.07			.09	

Note. *N* = 163. HB = health behavior. T_1_/T_2_ = first/second point of measurement. *B* = unstandardized regression coefficient. *SE* = standard error. *β* = standardized regression coefficient. UB = unhealthy behavior. HB at T_1_ corresponds to the behavior represented in each column. Social tie's influence for unhealthy behavior refers to the corresponding participant behavior. ^a^*p* < .05, ^b^*p* < .01.

### Living with parents and behavior/expectation change

Moving out predicted a significant increase in current and expected number of drinks as well as in current and expected binge drinking (Table 4). Moving out did not predict change in current or expected consumption of unhealthy food and physical inactivity. However, the effect of moving out was not mediated by pereceived parental and peer influences (*t*-scores of indirect effects ranged from −1.38 to 1.09, n.s.).

**Table 4.** Associations of having moved out of the parental home with students’ change in current and expected health behavior (regression analysis).

	Current food consumption (T_2_)			Current physical activity (T_2_)			Current # of drinks consumed monthly (T_2_)			Current binge drinking (T_2_)		
	*B*	*SE B*	*β*	*B*	*SE B*	*β*	*B*	*SE B*	*β*	*B*	*SE B*	*β*
HB at T_1_	.22	.07	.24^b^	–.27	.08	–.27^b^	.70	.13	.37^b^	.36	.07	.37^b^
Moved Out of Parents’ Home	–.08	.45	–.01	–.12	1.04	–.01	17.06	4.42	.27^b^	.45	.15	.22^b^
*R*^2^		.06^a^			.07^b^			.22^b^			.19^b^	

	Expected food consumption (T_2_)			Expected physical activity (T_2_)			Expected # of drinks consumed monthly (T_2_)			Expected binge drinking (T_2_)		
	*B*	*SE B*	*β*	*B*	*SE B*	*β*	*B*	*SE B*	*β*	*B*	*SE B*	*β*
Expected HB at T_1_	.13	.08	.14	–.26	.08	–.26^b^	.44	.18	.19^a^	.33	.07	.37^b^
Moved Out of Parents’ Home	–.37	.47	–.06	–.84	1.02	–.06	15.76	5.29	.23^b^	.35	.14	.18^a^
*R*^2^		.02			.07^b^			.10^b^			.17^b^	

Note. *N* = 163. HB = health behavior. T_1_/T_2_ = first/second point of measurement. *B* = unstandardized regression coefficient. *SE* = standard error. *β* = standardized regression coefficient. HB at T_1_ corresponds to the behavior represented in each column. Moving out of the parental home was represented as a dummy variable.

^a^ *p* < .05, ^b^*p* < .01.

## Discussion

Based on the ViolEx Model (Panitz et al., 2021), the present study investigated how reported health behaviors and expectations of first-year students change over their first semester, how the interplay between expectations and behavior relates longitudinally, and whether coping with expectation disconfirmation, social tie's efforts to motivate behavior, and moving out of the parental home relate to change in current and expected health behaviors. The first hypothesis, which assumed that first-year students’ health behaviors and expectations would become more negative throughout their first semester, was partially supported. Mean scores for current and expected number of drinks consumed per month increased while current and expected food consumption as well as current and expected physical activity became healthier over time. Findings regarding change in food consumption support the notion that high consumption of unhealthy food among first-year students appears to predominantly occur early in the first semester (i.e. within the first four months; Vadeboncoeur et al., 2015), highlighting the relevance of early prevention efforts. We speculate that these changes may occur as part of the process of independently establishing food consumption patterns (e.g. learning to prepare meals). Regarding change in physical activity, during the first measurement in November 2020, some participants may have been less physically active due closed fitness studios in Germany as per Covid-19 regulations (Zusammen gegen Corona, 2020) and limited opportunities for outdoor exercise due to cold weather. Thus, the observed increase in physical activity over time might be based on reopened fitness studios and more suitable weather conditions for outdoor exercise during the time of the second measurement in May 2021. The negative correlation between physical activity at the first and second measurement indicates that the initially most inactive students showed the strongest increase over time. Participants who were initially physically active despite closed fitness studios and bad weather were probably less affected by later change of these external conditions and therefore may have shown a different pattern of change in physical activity over time. Findings regarding change in alcohol use corroborate pre-pandemic reports (e.g. Prince et al., 2019) and suggest that, also during the Covid-19 pandemic, students’ alcohol use increased as they had opportunities to consume alcohol either alone or with others at places not affected by restrictions (e.g. dormitories, parks). Prince et al. (2019) found that students who reported greater alcohol use during the first semester showed greater escalation of drinking during the following years at university and greater Alcohol Use Disorder symptoms a year post graduation. Thus, reaching students early in their university career is an important protective strategy against post-university health risk behaviors.

While the second hypothesis on statistical effects of expectations on change in health behavior was not supported, we found partial support for predictive effects of the present health behavior on expectation change (Hypothesis 3) as initial physical activity predicted change in physical activity expectations, and initial alcohol use predicted change in alcohol-related expectations. Due to persistent Covid-19 restrictions (Zusammen gegen Corona, 2020), we speculate that it might have been difficult for participants to fulfill some of their expectations. Also, expectations may have been less realistic at the start of the first semester as many conditions were difficult to foresee. The predictive power of expectations may increase the more they are based on previous experiences. Our findings also indicate that previous experiences with alcohol can inform change in expectations so that differences between expected and actual consumption become smaller over time.

The present study represents the first longitudinal investigation relating the coping process of the ViolEx Model to health behaviors. We found partial support for the fourth hypothesis as ways of coping with expectation violation predicted change in expectations about physical activity, with those reporting to be active to fulfill their expectations being more likely to further increase their expectations and those who are more prone to ignoring and downplaying expectation-inconsistent information showing less expectation change. As such, the implementation of the ViolEx model has served to enhance our current understanding of the maintenance or change of physical-activity-related expectations, and, consistent with Panitz et al. (2021), findings suggest that individuals with higher assimilation and higher immunization would show less expectation change (Panitz et al., 2021). We speculate that coping processes may be more predictive of expectation change when also considering the direction of experienced expectation violation (i.e. whether reality is worse or better than expected; Panitz et al., 2021), and when testing how respondents cope with a specific expectation violation (i.e. specific event) rather than assessing coping in a general way. Interventions could benefit from assessing student's ways of coping with expectation violations and their current and expected physical activity levels to deliver tailored support based on knowledge on how they would cope if their expectations are disconfirmed. Although the present study addressed the gap in the literature concerning the maintenance or change of health-behavior-related expectations during the first year at university and found significant results regarding one domain (i.e. physical activity), future research should consider further investigating this topic as results may provide relevant support for interventions aimed at promoting healthy behavior.

The fifth hypothesis assumed that participants would change their behaviors and expectations to reflect their social ties’ influence based on reports suggesting that close social ties can affect an individual's behavior (Legros & Cislaghi, 2020) and expectations (Panitz et al., 2021). This hypothesis was partially supported as perceived initial parental attempts to inhibit physical activity and to promote alcohol use predicted increases over time in expected physical inactivity and in the number of drinks consumed by students per month. Our results indicate that parents play a significant role in their offspring's physical activity and alcohol use patterns during the first semester at university, which corroborates previous reports (Kaseva et al., 2017). As we did not specifically assess methods of motivating used by social ties, we can only speculate that parents may have conveyed permissiveness for and acceptance of unhealthy behaviors which first-year students may have interpreted as encouragements to behave and to expect to behave in unhealthy manners (Calhoun et al., 2018). Future studies should investigate what methods to encourage behavior are applied by social ties and how do these relate to changes in health behaviors. Still, significant results were not found regarding the influence of partners and peers on health behaviors. As the number of participants who reported having a romantic partner was low, additional statistical power might provide significant results. Regarding peers, participants could have had limited opportunities for joint health-related activities due to Covid-19 restrictions (Zusammen gegen Corona, 2020) which might have inhibited associations with perceived peer attempts to influence their behavior. Moreover, the transition to university is often characterized by instabilities in the peer network which reduces the predictive power of initial peer relations (Riordan & Carey, 2019).

Lastly, we hypothesized that housing would have an effect on participant's behaviors and expectations and that this effect would be mediated by parental and peer attempts to influence participant's behaviors. We found that moving out predicted a significant increase in current and expected alcohol use. Yet, statistical effects of leaving the parental home on changes in current and expected food consumption and physical activity were not found. We speculate that differences between living in the parental home and independent living might have been reduced in times of Covid-19 pandemic as students spent less time at the university town as most courses were conducted online. The effect of housing on alcohol use was not mediated by perceived parental and peer attempts to promote alcohol use. Thus, other processes may explain the observed statistical effect of housing on alcohol use, such as decline of parental monitoring after moving out (White et al., 2020) or influences of close friends, rather than of peers in general (Walther et al., 2017).

The current study presented several limitations. First, data was collected during the Covid-19 pandemic which presented unusual circumstances and restrictions (e.g. bars/pubs were closed at time of data collection, limited contact with other students at university) that reduced opportunities for some typically reported risk behaviors after the transition to university. As such, our results may not be applicable during times before and after the Covid-19 pandemic. We plan to follow-up on this limitation by repeating the study after Covid-19 restrictions are completely lifted. Second, females were overrepresented in our sample which could have affected our results. Future studies should include a more balanced gender distribution as well as a larger subject pool. Third, late data collection for the first point of measurement could have impacted the results as participants may have already been influenced by their peers and the university environment and their recall of past expectations and behaviors may have shifted to be more in-line with current behaviors and expectations. Lastly, we focused on investigating behavior changes during the first semester only as it is a crucial period for the development of unhealthy behaviors. Accordingly, our findings do not provide evidence about health behavior changes throughout the full university period. Future investigations could try to replicate our research among students of more advanced semesters to determine additional opportunities to prevent risk behaviors. Notwithstanding these limitations, our findings present important insights to improve health interventions for first-year students.

The observed different trajectories of food consumption and physical activity as well as of alcohol consumption indicate that the focus of early interventions related to these behaviors might differ. Regarding food consumption and physical activity, the focus might be to reduce levels of unhealthy behaviors observed early in the first semester whereas regarding alcohol use, a main focus should be to prevent increases in drinking. Reports from a systematic review and meta-analysis suggest the following intervention strategies to prevent unhealthy food consumption and physical inactivity among university students: addressing food consumption and physical activity separately, targeting self-efficacy, providing higher vigilance, encouragement, and support through frequent professional contact, and delivering feedback on student's progress rather than simply providing educational resources (Plotnikoff et al., 2015). Moreover, based on findings of a meta-analysis investigating the efficacy of alcohol interventions for first-year college students (Scott-Sheldon et al., 2014), interventions should combine several of the following intervention components to reduce alcohol intake and related problems among individuals: personalized feedback on consumption, problems, or risks, strategies to moderate drinking, challenges to alcohol-expectancies, and establishment of alcohol-related goals and limits. Lastly, interventions should address parental influence on physical activity and alcohol use as well as alcohol use among students that moved out of the parental home.

## Ethics statement

Institutional Review Board Statement: The study was conducted in accordance with the Declaration of Helsinki and was approved by an Institutional Review Board/Ethics committee. See details under Methods.

The study received an exemption from an Institutional Review Board/Ethics committee; See details under Methods.

## Supplementary Material

Supplemental Material
