# Supplementary material for: Influences on change in expected and actual health behaviors among first-year university students
Source: Health Psychol Behav Med. 2023 Feb 4;11(1):2174697. doi: 10.1080/21642850.2023.2174697 (PMC9901434; doi:10.1080/21642850.2023.2174697)
Supplement: Supplemental Material [file RHPB_A_2174697_SM2355.docx]

**Supplementary Material**

**S1.** ***Social Ties’ Efforts to Encourage Health Risk Behaviors (Self-Developed Items).***

1. How often do your peers encourage you to consume unhealthy foods?

- Very often
- Often
- Seldom
- Never

1. How often do your peers encourage you to drink a lot of alcohol?

- Very often
- Often
- Seldom
- Never

1. How often do your peers encourage you to not be physically active?

- Very often
- Often
- Seldom
- Never

1. How often do your parents encourage you to consume unhealthy foods?

- Very often
- Often
- Seldom
- Never

1. How often do your parents encourage you to drink a lot of alcohol?

- Very often
- Often
- Seldom
- Never

1. How often do your parents encourage you to not be physically active?

- Very often
- Often
- Seldom
- Never

1. How often does your romantic partner encourages you to consume unhealthy foods?^1^

- Very often
- Often
- Seldom
- Never

1. How often does your romantic partner encourages you to drink a lot of alcohol? ^1^

- Very often
- Often
- Seldom
- Never

1. How often does your romantic partner encourage you to not be physically active? ^1^

- Very often
- Often
- Seldom
- Never

**Supplementary Material**

**S2***.* ***Comparison of Completers and Dropouts.***

|  | *Completers* | | *Drop-Outs* | |  |  |  |
| --- | --- | --- | --- | --- | --- | --- | --- |
|  | *M_1_* | *SD_1_* | *M_1_* | *SD_1_* | *t* | *df* | *p* |
| **Demographics** |  |  |  |  |  |  |  |
| Age | 20.71 | 2.68 | 20.47 | 2.58 | –.62 | 206 | .54 |
| Sex | 1.83 | .41 | 1.87 | .41 | .48 | 206 | .64 |
| Hometown | 1.05 | .22 | 1.00 | .01 | –1.32 | 206 | .19 |
| **Behavior** |  |  |  |  |  |  |  |
| FC | 19.64 | 2.59 | 19.63 | 2.76 | –.03 | 206 | .97 |
| PA | 24.42 | 5.86 | 22.85 | 5.79 | –1.61 | 206 | .11 |
| ND | 9.78 | 15.31 | 11.00 | 14.44 | .50 | 206 | .62 |
| BD | 1.89 | .91 | 2.01 | .94 | .75 | 206 | .46 |

*Note. FC* = food consumption; *PA* = physical activity; *ND* = number of drinks consumed per month; *BD* = binge drinking; *M_1_* = mean score at time 1; *SD_1_* = standard deviation at time 1; *t* = test of between-group differences; *df* = degrees of freedom.

**S3. *Bivariate Correlations Between All Model Variables.***

|  | ***M (SD)*** | **1** | **2** | **3** | **4** | **5** | **6** | **7** | **8** | **9** | **10** | **11** | **12** | **13** | **14** | **15** | **16** |
| --- | --- | --- | --- | --- | --- | --- | --- | --- | --- | --- | --- | --- | --- | --- | --- | --- | --- |
| **Behavior** |  |  |  |  |  |  |  |  |  |  |  |  |  |  |  |  |  |
| 1. FC-1 | 19.63 (2.72) | 1.00 | .14* | 0.05 | 0.10 | .24** | -0.07 | 0.00 | -0.05 | .62** | -0.07 | -0.01 | -0.02 | 0.12 | -0.08 | -0.04 | -0.08 |
| 2. PA-1 | 23.19 (5.83) | .14* | 1.00 | -0.03 | 0.06 | -0.15 | -.27** | 0.01 | 0.04 | -0.03 | .78** | -0.03 | -0.03 | -0.15 | -.29** | 0.05 | -0.01 |
| 3. ND-1 | 10.74 (14.61) | 0.05 | -0.03 | 1.00 | .69** | 0.04 | 0.12 | .38** | .29** | .23** | 0.00 | .76** | .53** | 0.13 | 0.12 | .29** | .34** |
| 4. BD-1 | 1.98 (.93) | 0.10 | 0.06 | .69** | 1.00 | 0.13 | 0.06 | .36** | .38** | .16* | -0.01 | .56** | .77** | 0.14 | 0.04 | .34** | .43** |
| 5. FC-2 | 15.72 (2.57) | .24** | -0.15 | 0.04 | 0.13 | 1.00 | 0.13 | 0.08 | 0.15 | .17* | -0.15 | 0.00 | 0.11 | .79** | 0.11 | 0.08 | 0.08 |
| 6. PA-2 | 13.88 (5.96) | -0.07 | -.27** | 0.12 | 0.06 | 0.13 | 1.00 | 0.13 | 0.14 | 0.02 | -.18* | 0.13 | 0.10 | .16* | .81** | .17* | 0.09 |
| 7. ND-2 | 25.03 (27.68) | 0.00 | 0.01 | .38** | .36** | 0.08 | 0.13 | 1.00 | .66** | 0.12 | 0.03 | .32** | .35** | 0.09 | 0.12 | .68** | .65** |
| 8. BD-2 | 1.97 (.90) | -0.05 | 0.04 | .23** | .38** | 0.15 | 0.14 | .66** | 1.00 | -0.05 | 0.04 | .27** | .34** | 0.09 | 0.05 | .54** | .77** |
| **Expectation** |  |  |  |  |  |  |  |  |  |  |  |  |  |  |  |  |  |
| 9. FC-1 | 19.23 (2.72) | .62** | -0.03 | .23** | .16* | .17* | 0.02 | 0.12 | -0.05 | 1.00 | 0.05 | .16* | 0.09 | 0.14 | 0.05 | 0.01 | 0.07 |
| 10. PA-1 | 21.34 (5.68) | -0.07 | .78** | 0.00 | -0.01 | -0.15 | -.18* | 0.03 | 0.04 | 0.05 | 1.00 | -0.01 | -0.05 | -0.14 | -.25** | 0.05 | 0.00 |
| 11. ND-1 | 11.01 (13.15) | -0.01 | -0.03 | .76** | .56** | 0.00 | 0.13 | .32** | .27** | .16* | -0.01 | 1.00 | .56** | 0.06 | 0.14 | .21** | .27** |
| 12. BD-1 | 1.90 (.93) | -0.02 | -0.03 | .53** | .77** | 0.11 | 0.10 | .35** | .34** | 0.09 | -0.05 | .56** | 1.00 | 0.12 | 0.11 | .26** | .38** |
| 13. FC-2 | 15.50 (2.63) | 0.12 | -0.15 | 0.13 | 0.14 | .79** | .16* | 0.09 | 0.09 | 0.14 | -0.14 | 0.06 | 0.12 | 1.00 | .27** | 0.05 | .16* |
| 14. PA-2 | 15.91 (5.84) | -0.08 | -.29** | 0.12 | 0.04 | 0.11 | .81** | 0.12 | 0.05 | 0.05 | -.25** | 0.14 | 0.11 | .27** | 1.00 | 0.10 | 0.14 |
| 15. ND-2 | 22.51 (30.68) | -0.04 | 0.05 | .29** | .34** | 0.08 | .17* | .68** | .54** | 0.01 | 0.05 | .21** | .26** | 0.05 | 0.10 | 1.00 | .56** |
| 16. BD-2 | 1.89 (.85) | -0.08 | -0.01 | .34** | .43** | 0.08 | 0.09 | .65** | .77** | 0.07 | 0.00 | .27** | .38** | .16* | 0.14 | .56** | 1.00 |

*Note. FC* = food consumption; *PA* = physical activity; *ND* = number of drinks consumed per month; *BD* = binge drinking; *-1* = time 1; *-2* = time 2; *M* = mean score; *SD* = standard deviation.

* *p* < .05

** *p* < .01
